# Description of future drought indices in Virginia

**DOI:** 10.1016/j.dib.2017.07.042

**Published:** 2017-07-20

**Authors:** Hyunwoo Kang, Venkataramana Sridhar

**Affiliations:** Biological Systems Engineering Department (MC0303), Virginia Tech, 155 Ag Quad Lane, Blacksburg, VA 24061, United States

**Keywords:** Drought, Modeling, Climate change, Virginia, USA

## Abstract

This article presents projected future drought occurrences in five river basins in Virginia. The Soil and Water Assessment Tool (SWAT) and the Coupled Model Intercomparison Project Phase 5 (CMIP5) climate models were used to derive input variables of multiple drought indices, such as the Standardized Soil Moisture index (SSI), the Multivariate Standardized Drought Index (MSDI), and the Modified Palmer Drought Severity Index (MPDSI) for both historic and future periods. The results of SSI indicate that there was an overall increase in agricultural drought occurrences and that these were caused by increases in evapotranspiration and runoff. However, the results of the MSDI and MPDSI projected a decrease in drought occurrences in future periods due to a greater increase in precipitation in the future. Furthermore, GCM-downscaled products (precipitation and temperature) were verified using comparisons with historic observations, and the results of uncertainty analyses suggest that the lower and upper bounds of future drought projections agree with historic conditions.

## Specification Table

**Table:** 

Subject area	Environmental Science
More specific subject area	Climate change impacts on drought occurrences
Type of data	Figures and Tables
How data were acquired	Data were acquired using hydrological modeling with the Soil and Water Assessment Tool (SWAT) model and Coupled Model Intercomparison Project Phase 5 (CMIP5) climate scenarios
Data format	Analyzed
Data source location	Downscaled CMIP5 precipitation and temperature data was from http://gdo-dcp.ucllnl.org.
Experimental factors	R coding was used to compute multiple drought indices.
Experimental features	Standardized Soil Moisture index (SSI), Multivariate Standardized Drought Index (MSDI), and Modified Palmer Drought Severity Index (MPDSI) for both historic (his: 1970−1999) and future periods (f1: 2020−2049, f2: 2050−2079) were computed.
Data accessibility	The data are available in this article.

## Data value

● Provides information about the spatio-temporal patterns of future drought occurrences and seasonal characteristics.
● Can be used to identify areas vulnerable to climate change and droughts.
● Can contribute significantly to research in the fields of drought risk management and drought mitigation strategies.

## 1 Data

### 1.1 Drought indices

**Fig. 1:**
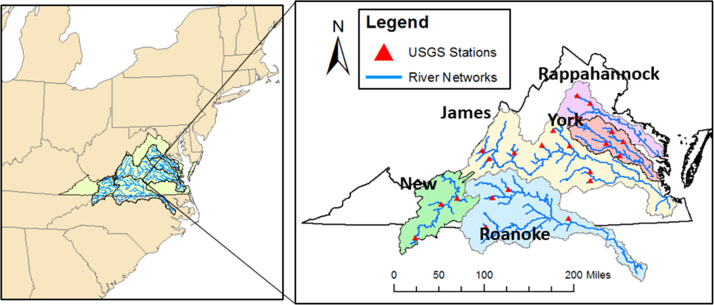
Location map of the five river basins in Virginia. The red triangles represent the USGS stream gauge stations, and the blue lines represent the river networks.

The figures and tables in this article provide analyses of drought indices computed with the Soil and Water Assessment Tool [1] and Coupled Model Intercomparison Project Phase 5 (CMIP5) climate model [6]. Fig. 1 shows the location of five main river basins in Virginia; they are the James, New, Rappahannock, Roanoke, and York River basins. Additionally, Table 1 shows the CMIP5 climate models that were used in this article.

**Table 1:** List of CMIP5 models.

Abbreviation	Name
S1	access1-0
S2	bcc-csm1-1.1
S3	canesm2
S4	ccsm4
S5	cesm1-bgc.1
S6	cnrm-cm5.1
S7	csiro-mk3-6-0
S8	gfdl-esm2g.1
S9	inmcm4
S10	ipsl-cm5a-mr

**Fig. 2:**
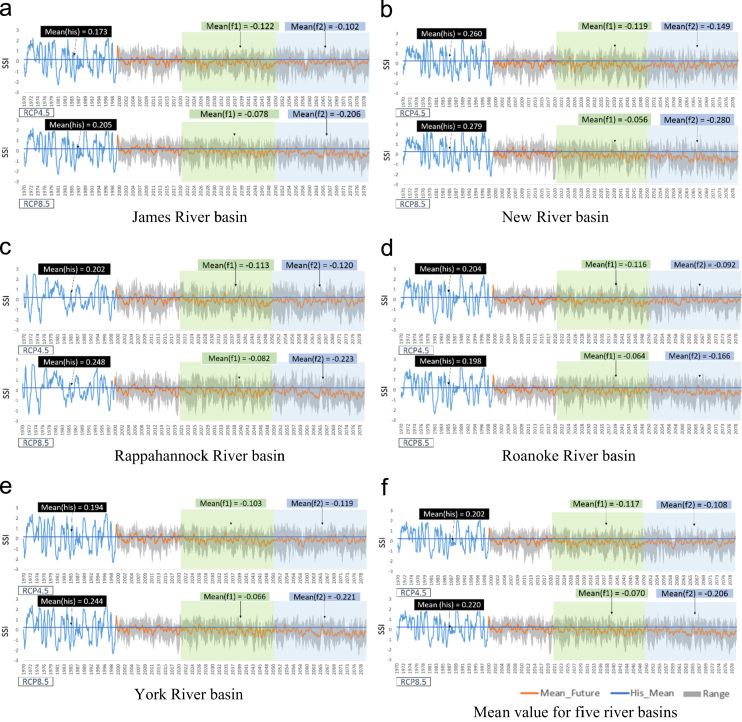
Weekly time series of SSI for each river basin. The blue lines indicate the SSI values for the historic period, and the orange lines are the mean values of the future period from ten climate models. Transparent green and blue rectangles highlight the future periods.

**Fig. 3:**
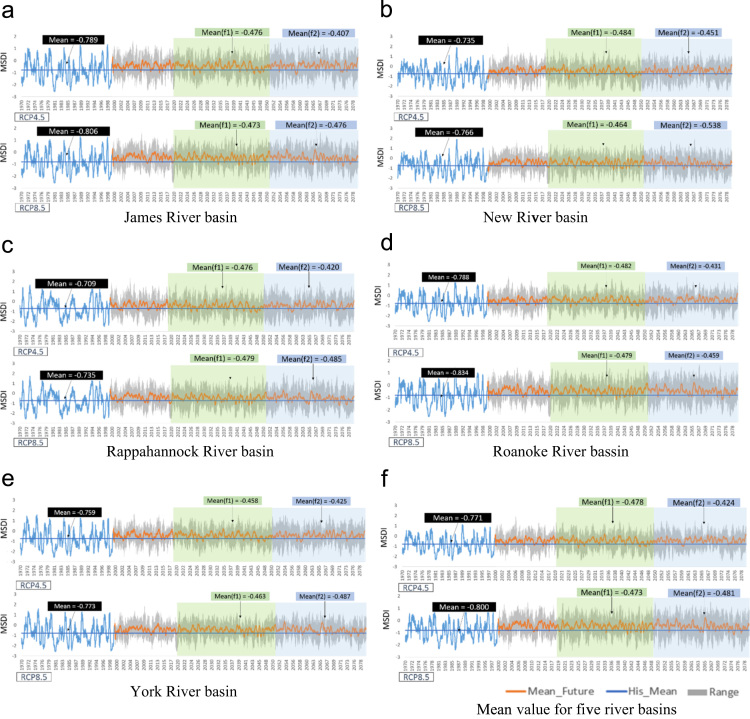
Weekly time series of MSDI in each river basin. The blue lines represent the MSDI values for the historic period, and the orange lines are the mean values for the future period from ten climate models. Transparent green and blue rectangles highlight the future periods.

**Fig. 4:**
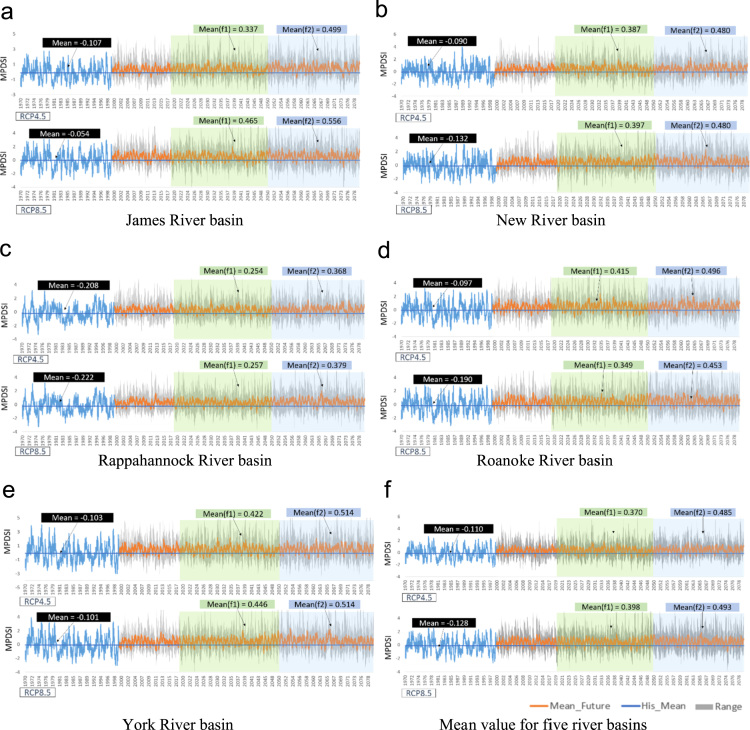
Weekly time series of MPDSI for each river basin. The blue lines represent the MPDSI values for the historic period, and the orange lines are the mean values for the future period from ten climate models. Transparent green and blue rectangles highlight the future periods.

Fig. 2 shows the weekly time series of the Standardized Soil Moisture index (SSI; [4]), and the Modified Palmer Drought Severity Index (MPDSI, [5]), which were based on SWAT outputs such as soil moisture, runoff, and evapotranspiration (ET). In these figures, blue straight lines represent the historical mean value of drought indices, and orange lines represent the ensemble mean values of the drought indices for projected periods. Additionally, the gray areas show the range of the climate models (minimum to maximum). The mean values of SSI for the historic period were 0.202 and 0.220 for RCP 4.5 and RCP 8.5. However, the mean values for future periods were −0.117 and −0.108 for RCP 4.5, and −0.070 and −0.206 for RCP 8.5, respectively. Overall, there was long-term increase in agricultural drought occurrences in the future. Fig. 3 represents the weekly time series of the Multivariate Standardized Drought Index (MSDI, [4]) as an overall average and for each of the basins. The mean values of MSDI for the historic period were −0.771 and −0.800 for RCP 4.5 and RCP 8.5. However, the mean values for the future periods were −0.478 and −0.424 for RCP 4.5, and −0.070 and −0.206 for RCP 8.5, respectively. In contrast, with the results of SSI, the mean values of MSDI for the future periods were larger than the historic means of MSDI. Thus, the results implied that there was a long-term decrease in the multivariate perspective of droughts in the future. Fig. 4 shows the weekly time series of the Modified Palmer Drought Severity Index (MPDSI, [5]) as an overall average and for each of the basins. The mean values of MPDSI for the historic period were −0.110 and −0.128 for RCP 4.5 and RCP 8.5. Additionally, the mean values for the future periods were 370 and 0.485 for RCP 4.5, and 0.398 and 0.493 for RCP 8.5, respectively. Similar to the results of MSDI, the mean values of MPDSI in the future periods were larger than the historic means of MPDSI. Thus, the results projected a long-term decrease in meteorological droughts in the future.

**Fig. 5:**
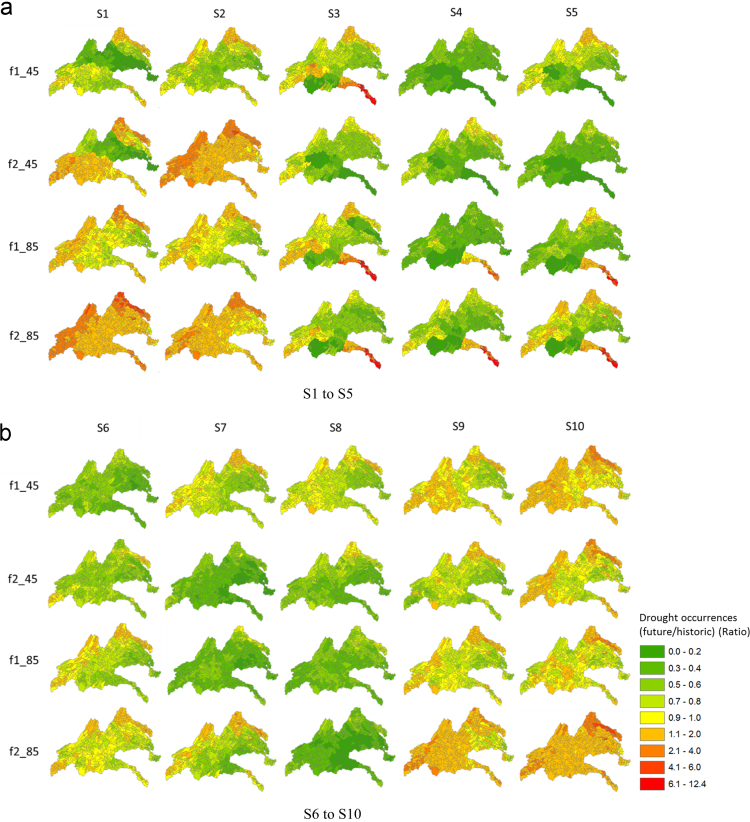
Spatial maps of the comparisons of drought occurrences based on the results of SSI between historic and future periods. f1_45 represents the result of RCP 4.5 in the f1 period, f2_45 represents RCP 4.5 in the f2 period.

**Fig. 6:**
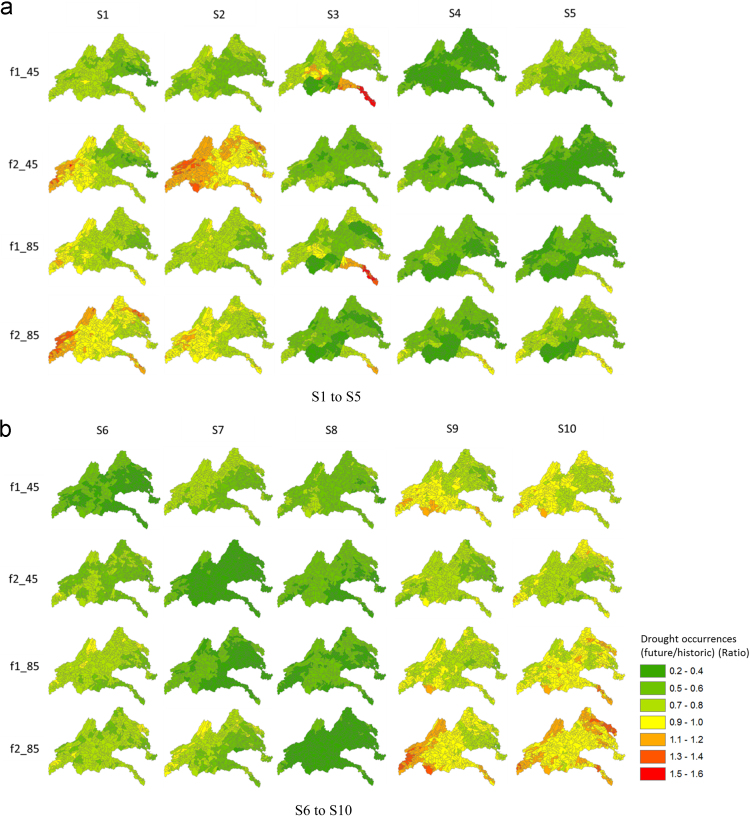
Spatial maps of the comparisons of drought occurrences based on results of MSDI between historic and future periods. f1_45 represents the result of RCP 4.5 in the f1 period, f2_45 represents RCP 4.5 in the f2 period.

**Fig. 7:**
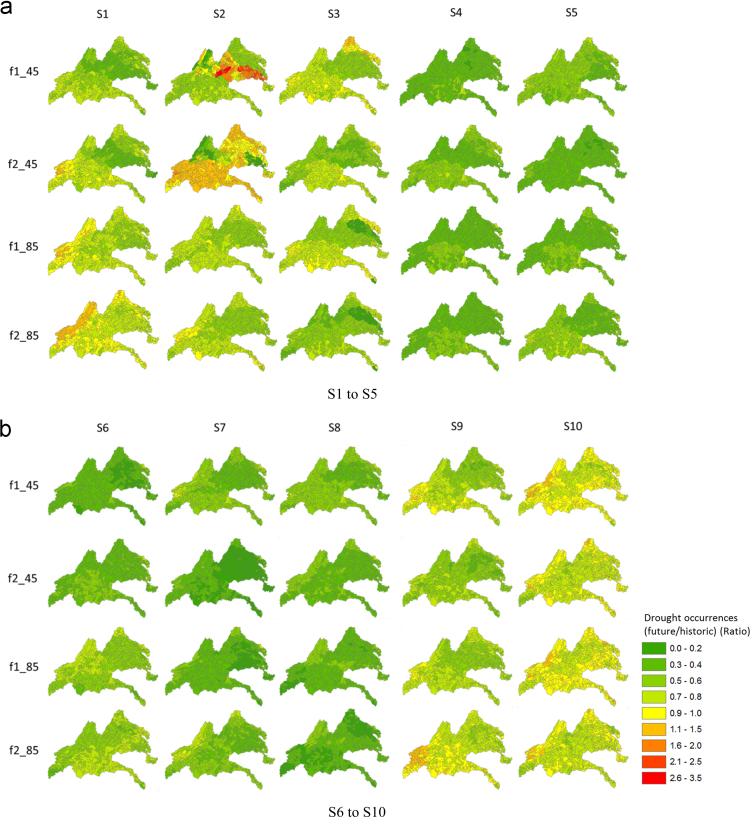
Spatial maps of the comparisons of drought occurrences based on the results of MPDSI between historic and future periods. f1_45 indicates the results of RCP 4.5 in the f1 period, f2_45 is RCP 4.5 in the f2 period.

Fig. 5 provides spatial maps of the ratio of drought occurrences based on SSI for the five river basins. A value equal or greater than 1 indicates an increase in drought occurrences in the future; other values less than 1 indicate a decrease in drought occurrences in the future. As shown in Fig. 5, there was an overall increase in drought occurrences in the future during both the f1 and f2 periods in several climate models, such as S1, S2, S9, and S10 for both RCP 4.5 and RCP 8.5. From these results, it can be said that the New and Rappahannock river basins are vulnerable to agricultural droughts among the five river basins. Fig. 6 provides spatial maps of the ratio of drought occurrences based on the MSDI for the five river basins. As shown in this figure, there was an overall decrease in drought occurrences for most climate models, whereas several areas increased in S1 and S2 in the New River basin during the second future period (f2). The MSDI was proposed to consider multivariate variables (precipitation and soil moisture) for drought evaluation, and it is known that the onset and termination of droughts are influenced by both precipitation and soil moisture. Thus, the drought occurrences derived by MSDI were different from SSI, which was influenced by increases in precipitation in the future. Fig. 7 provides spatial maps of the ratio of drought occurrences based on MPDSI in the five river basins, and there was an overall decrease in drought occurrences in the future during both the f1 and f2 periods. Since MPDSI is a meteorological drought index, it can be said that drought occurrences estimated by MPDSI were influenced by projected increases in precipitation in the future.

### 1.2 Verification of GCM performance

**Fig. 8:**
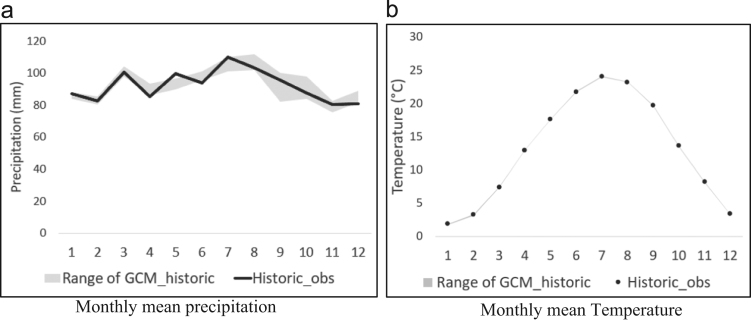
Comparison of historic observation and GCM_historic (1950–1999) precipitation and temperature. (a) The black solid line represents the monthly mean values of precipitation for historic observation, and the gray line is the range of GCM_historic. (b) The black dots indicate the monthly mean values of temperature for historic observation.

To verify the performance and accuracy of multiple global climate models (GCMs) of CMIP5, comparisons of historic observations and GCM-downscaled historic precipitation and temperature should be performed. Fig. 8 provides a comparison of historic observations and a range of GCM-downscaled historic data for the five river basins in Virginia (mean value of 480 grids). Additionally, Table 2, Table 3 show the monthly differences in precipitation and temperature between historic observations and GCM-downscaled historic observations. The greatest difference in precipitation as 10.4% in the S1 model in September, and for temperature, it was −0.11 °C in the S1 model in April. Overall, the GCM-downscaled products agreed well with historic observations.

**Table 2:** Differences in monthly mean precipitation between the historic observations and GCM_historic (Unit: %).

Month	S1	S2	S3	S4	S5	S6	S7	S8	S9	S10
Jan	−0.4	1.1	1.0	−0.4	3.2	1.6	0.2	−1.0	−1.0	−0.6
Feb	0.9	−1.2	2.6	0.7	−3.5	−3.6	−3.1	2.3	−1.2	2.7
Mar	1.2	−0.2	−3.3	−2.4	0.9	2.7	2.1	0.0	−0.9	−2.9
Apr	−9.4	−2.1	−1.2	−1.7	−4.7	−5.6	−4.1	−4.5	−2.0	−3.2
May	9.9	2.8	4.9	4.8	5.5	5.8	3.1	2.9	4.4	6.4
Jun	−7.2	−5.8	−5.1	−2.5	−3.1	−6.7	−1.6	−1.6	−2.9	−7.7
Jul	4.9	6.6	2.8	−0.1	1.9	5.8	2.7	3.2	0.7	8.0
Aug	−5.0	1.1	−1.7	0.4	−2.1	−0.2	0.4	−2.5	−0.2	−8.4
Sep	10.4	−4.8	9.1	7.9	7.8	0.2	1.7	5.7	5.4	10.1
Oct	−5.7	4.0	−8.5	−6.4	−3.8	1.5	0.6	−5.6	−2.9	−10.0
Nov	0.9	−1.3	6.4	2.4	0.5	−2.2	−2.8	3.4	−0.6	5.9
Dec	−5.0	−2.9	−10.2	−4.3	−5.6	−1.1	−1.1	−4.1	−1.5	−5.9

**Table 3:** Differences in the monthly mean precipitation between the historic observations and GCM_historic (Unit: °C).

Month	S1	S2	S3	S4	S5	S6	S7	S8	S9	S10
Jan	0.02	0.03	0.06	0.04	0.03	0.03	0.10	0.01	0.05	0.12
Feb	0.05	0.07	0.07	0.02	0.05	0.06	0.14	0.05	−0.05	0.07
Mar	0.05	0.02	0.02	0.04	0.04	0.03	0.05	0.01	0.02	−0.04
Apr	−0.11	−0.02	0.00	−0.08	−0.04	−0.03	−0.02	−0.10	−0.10	−0.06
May	−0.03	−0.03	−0.06	−0.06	−0.06	−0.07	−0.02	−0.05	−0.08	−0.08
Jun	−0.01	−0.08	−0.04	0.02	0.01	−0.03	−0.04	−0.05	0.02	0.02
Jul	−0.07	−0.06	−0.05	0.02	0.01	−0.04	−0.03	−0.05	0.02	−0.04
Aug	−0.01	−0.05	−0.02	0.01	0.01	−0.03	−0.02	0.01	−0.01	0.01
Sep	0.01	−0.02	0.00	0.01	0.03	−0.01	0.00	−0.01	−0.05	0.05
Oct	0.06	0.06	0.03	0.09	0.06	0.00	0.01	−0.03	0.06	0.11
Nov	−0.02	0.07	0.05	0.08	0.03	0.08	0.09	0.00	0.01	0.01
Dec	0.05	0.05	0.03	0.07	0.06	0.06	0.04	0.02	0.05	0.05

### 1.3 Parameter uncertainty in drought projections

**Fig. 9:**
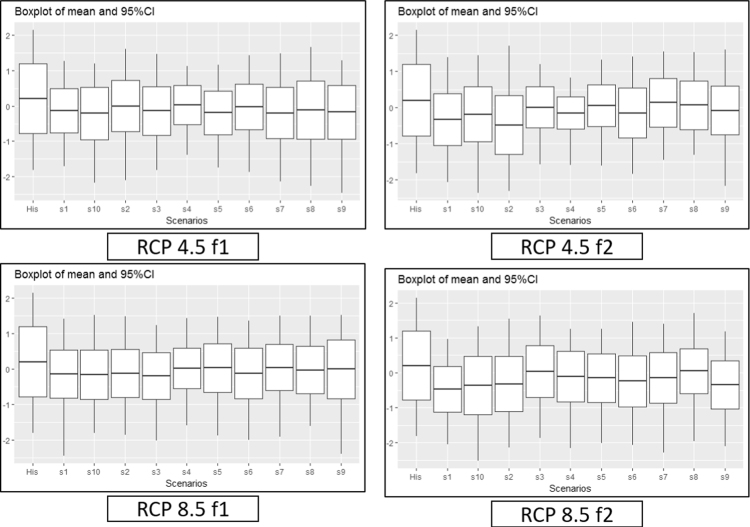
A boxplot of the mean and 95% confidence intervals for historic and future SSI in the five river basins. The left box plot represents the historic SSI for the historic period, and the other box plots represent the future period for each climate model.

**Fig. 10:**
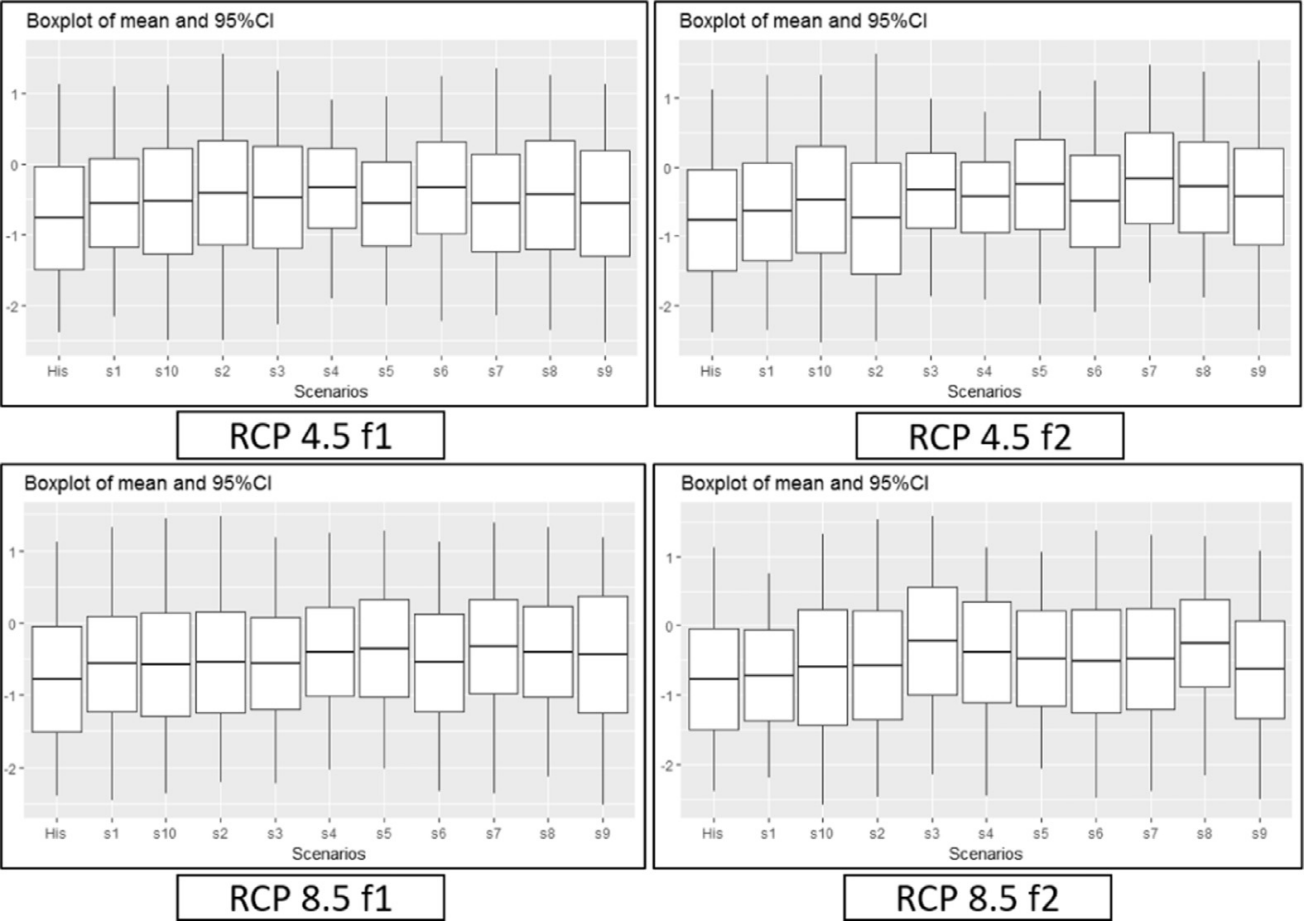
Boxplot of the mean and 95% confidence interval for the historic and future MSDI in the five river basins. The left box plot represents the historic MSDI in the historic period, and the other box plots represent the future period for each climate model.

**Fig. 11:**
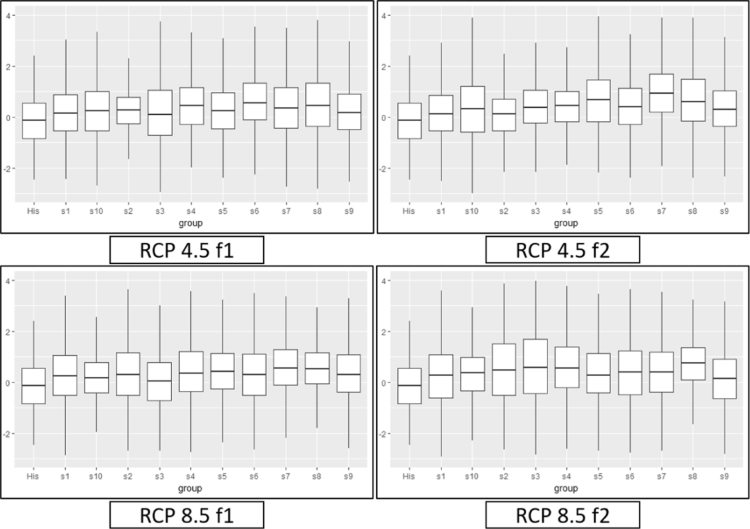
Boxplot of the mean and 95% confidence intervals for historic and future MPDSI for five river basins. The left box plot represents the historic MPDSI for the historic period, and the other box plots represent the future period for each climate model.

Fig. 9, Fig. 10, Fig. 11 provide a boxplot of the mean and 95% confidence interval of each drought index in the five river basins (historic and future periods). In the case of SSI, the overall decrease in the future occurred in both RCP 4.5 and RCP 8.5 during the f1 and f2 periods, while MSDI and MPDSI showed an increase in mean values. The parameter uncertainty in capturing the historic index was verified with distribution and confidence intervals. The uncertainty that exists in the model that derived the SSI, MSDI, and MPDSI was higher or equal to the future drought indices, which suggests that the lower and upper bounds of future projects are somewhat similar to the historic conditions.

### 1.4 Temporal shift in soil moisture

**Fig. 12:**
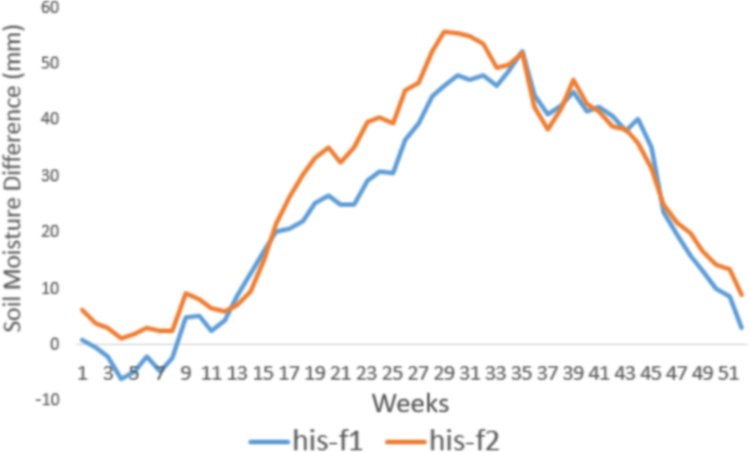
Soil moisture differences between historic and future periods. The orange line represents the difference between the historic and f1 period, and the blue line is the difference between the historic and f2 period.

Fig. 12 provides the soil moisture differences between historic and future periods (f1 and f2) for the New river basin for the S2 model, and it indicates that increased drought based on SSI could be directly related to a soil moisture deficit.

## 2 Materials and methods

In this article, SWAT was used to estimate the input variables for multiple drought indices in the evaluation of future droughts. For example, soil moisture derived by SWAT was used for computation of the SSI, MSDI, and MPDSI. Additionally, ET, potential ET, and runoff were also used to calculate MPDSI.

The joint probability method with joint behavior of two random variables (*X* and *Y*) was used to compute MSDI, and the joint distribution of two variables can be expressed as follows:

(1) P(X≤x,Y≤y)=p

where *p* is the joint probability of the precipitation and soil moisture. Additionally, MSDI can be defined as follows [3]:

(2) $$\mathrm{MSDI}=\emptyset^{-1}(p)$$

where ∅ is the standard normal distribution function.

In this article, an alternative methodology based on an empirical joint probability was used, which was the Grigorten plotting position formula [2]:

(3) $$P\left(x_{k},y_{k}\right)=\frac{m_{k}-0.44}{n+0.12}$$

where $m_{k}$ is the number of occurrences of the pair ($x_{i},y_{i}$) for $x_{i}\leq x_{k}$ and $y_{i}\leq y_{k}$, and n is the number of the observation. For the computation of SSI, a univariate form of the Gringorten plotting position formula (Eq. (5)) [2] was also used:

(4) $$P\left(x_{i}\right)=\frac{i-0.44}{n+0.12}$$

where n is the number of observations i is the rank of the measured values from the smallest.

Furthermore, MPDSI [5] was used for evaluation of future drought conditions. Historic and future MPDSI values were computed using the input and output from SWAT, which were as follows: precipitation, potential and actual evapotranspiration, soil moisture, and runoff. MPDSI is based on the water balance equation and the adjustment between the actual and climatological estimation known as “Climatically appropriate for existing conditions (CAFEC)”:

(5) d=P−CAFEC,where

(6) CAFEC=αPE+βPR+γPRO+δPL

where PE is the potential evapotranspiration, PR is the potential recharge, PRO is the potential runoff, and PL is the potential soil moisture loss, and the coefficient α,β,γ,andδ are the ratios of the mean variables. In this article, weekly based SSI, MSDI (25-week scale), and MPDSI were computed based on input and output variables from the SWAT model.

## References

[1] Arnold J.G., Srinivasan R., Muttiah R.S., Williams J.R. (1998). Large area hydrologic modeling and assessment Part I: model development1. J. Am. Soc. Water Resour. Assoc..

[2] Gringorten I.I. (1963). A plotting rule for extreme probability paper. J. Geophys. Res..

[3] Hao Z., AghaKouchak A. (2013). Multivariate standardized drought index: a parametric multi-index model. Adv. Water Resour..

[4] Hao Z., AghaKouchak A. (2014). A nonparametric multivariate multi-index drought monitoring framework. J. Hydrometeorol..

[5] Mo K.C., Chelliah M. (2006). The modified Palmer drought severity index based on the NCEP North American Regional Reanalysis. J. Appl. Meteorol. Climatol..

[6] Taylor K.E., Stouffer R.J., Meehl G.A. (2012). An overview of CMIP5 and the experiment design. Bull. Am. Meteorol. Soc..

